# Kaposi's sarcoma responding to topical imiquimod 5% cream: a case report

**DOI:** 10.4076/1757-1626-2-7092

**Published:** 2009-09-17

**Authors:** Sophia Benomar, Saber Boutayeb, Leila Benzekri, Hassan Errihani, Badreddine Hassam

**Affiliations:** 1 Department of Dermatology, Ibn Sina hospital, Rabat, Morocco; 2 Department of Medical Oncology, National Oncology Institute, Rabat, Morocco

## Abstract

Kaposi's sarcoma, a virus-associated neoplasm, can be treated with systemic therapy such interferon or chemotherapy, although a local alternative is possible in localized disease.

Topical imiquimod is a ligand of the toll-like receptors 7 and 8 on dendritic cells, increasing immune responses and showing antiviral and antitumoral activities. We report a spectacular response to imiquimod 5% cream in a patient with classic Kaposi's sarcoma of the leg.

## Introduction

Kaposi's sarcoma (KS) is a vascular neoplasm with four clinical subtypes: classic, African immunosuppression-associated and AIDS-related. All variants have been related to the eighth human herpes virus (HHV8) [1].

Interferon alpha was used at high dose in AIDS-related KS and low-dose in classic and endemic KS [2]. Therefore, topical imiquimod, an immune response modifier with antiangiogenic properties able to induce interferon-alpha secretion in situ, could prove a good local treatment for KS skin lesions [3].

## Case presentation

A 57-year-old HIV negative Mediterranean man was treated with imiquimod 5% cream for purple maculo-papular cutaneous lesions on the leg that were histologically consistent with KS (papillary dermal proliferation of small angulated vessels lined by bland endothelial cells with an accompanying sparse infiltrate of lymphocytes and plasma cells).

The treatment protocol consisted of 3 weekly applications (Monday, Thursday, Saturday) under occlusion for at least 10 hours (between 10 and 12 hours). During the first week, the three applications were applied by the patient under supervision then the cream was self-administered for the following applications. The tolerance was good with no side effects. After twenty weeks, the purple maculo-papular lesions cleared (complete clinical response). Six months later the patient is still disease free.

## Discussion

Imiquimod 5% cream is a topically active immune response modifier approved as a treatment for actinic keratosis, superficial basal cell carcinoma, and external genital warts. [4] The drug up-regulates cytokine production, such as alpha-interferon, tumor necrosis factor alpha and promotes a T-helper type 1 cell-mediated immune response. Adverse events reported with imiquimod treatment include local side effects at the application site, ranging from mild erythema to weeping, crusting, and erosions. Systemic side effects are exceptional [5].

Concerning the literature, there are only two cases reports of KS treated by imiquimod and one phase II study. In the first report, the KS was AIDS-related whereas the second was a classic Kaposi's sarcoma [6,7].

In this last report, the patient was treated for classic Kaposi's sarcoma of both legs with imiquimod 5% cream once daily after progression under etoposide at the dose of 50 mg/day. After one year of therapy a complete response was observed and confirmed according to histopathology and immunohistochemistry.

The treatment was well tolerated, and the only significant side effects were flu-like symptoms that disappeared with the reduction of the dosage. After more than one year of follow-up, the patient was still disease-free. The tolerance was good with only transient itching and flu-like symptoms.

The phase II trial was conducted by a French team, and 17 patients were enrolled for classic or endemic KS. The treatment by imiquimod 5% cream was applied under occlusion three times per week for 24 weeks. Eight patients (47%) presented objective overall clinical response (2 complete and 6 partial responses). Tumor progression was noted in 6 patients. The most frequent side effects were local itching and erythema, seen in 9 patients (53%) [8].

For all the patients, there were no clinically meaningful changes from baseline in any of the laboratory test values, physical examinations, or vital sign measurements during the course of the study. There were no treatment-related deaths or serious adverse events.

Immunocompetent patients with new and small skin lesions seem to be ideal patients for treatment by topical imiquimod. Imiquimod cream offers an attractive alternative to locally destructive therapies, with less risk of pain, ulceration, and residual scarring. Furthermore, imiquimod can be applied by the patient.

## Conclusion

Topical imiquimod seems to be a good local treatment for Kaposi's sarcoma skin lesions with a good toxicity profile, regimens with daily application may offer best clinical results and should be explored and evaluated concerning the toxicity.

## Consent

Written informed consent was obtained from the patient for the publication of this case. A copy of the written consent is available for review by the Editor-in-Chief of this journal.

## Competing interests

The authors have indicated no significant interest with commercial supporters.

## Authors' contributions

SB and BS interpreted the patient data regarding the disease history and contributed to the literature research. BL and BH and HE contributed to the treatment of the disease and reviewed the manuscript.
